# Disturbed Interhemispheric Functional Connectivity Rather than Structural Connectivity in Irritable Bowel Syndrome

**DOI:** 10.3389/fnmol.2016.00141

**Published:** 2016-12-06

**Authors:** Rongfeng Qi, Chang Liu, Yifei Weng, Qiang Xu, Liya Chen, Fangyu Wang, Long J. Zhang, Guang M. Lu

**Affiliations:** ^1^Department of Medical Imaging, Jinling Hospital, Medical School of Nanjing UniversityNanjing, China; ^2^Department of Gastroenterology, Jinling Hospital, Medical School of Nanjing UniversityNanjing, China

**Keywords:** irritable bowel syndrome, interhemispheric voxel-mirrored homotopic connectivity, resting-state, functional connectivity, magnetic resonance imaging

## Abstract

Neuroimaging studies have demonstrated that irritable bowel syndrome (IBS)—a relapsing functional bowel disorder—presents with disrupted brain connections. However, little is known about the alterations of interhemispheric functional connectivity and underlying structural connectivity in IBS. This study combined resting-state functional magnetic resonance imaging (rs-fMRI) and diffusion tensor imaging (DTI) to investigate changes in interhemispheric coordination in IBS patients. Resting-state functional and structural magnetic resonance images were acquired from 65 IBS patients and 67 healthy controls (HCs; matched for age, sex and educational level). Interhemispheric voxel-mirrored homotopic connectivity (VMHC) was calculated and compared between groups. Homotopic regions showing abnormal VMHC in patients were targeted as regions of interest (ROIs) for analysis of DTI tractography. The fractional anisotropy (FA), fiber number and fiber length were compared between groups. Statistical analysis was also performed by including anxiety and depression as covariates to evaluate their effect. A Pearson correlation analysis between abnormal interhemispheric connectivity and clinical indices of IBS patients was performed. Compared to HCs, IBS patients had higher interhemispheric functional connectivity between bilateral thalami, cuneus, posterior cingulate cortices (PCC), lingual gyri and inferior occipital/cerebellum lobes, as well as lower interhemispheric functional connectivity between bilateral ventral anterior cingulate cortices (vACC) and inferior parietal lobules (IPL). The inclusion of anxiety and depression as covariates abolished VMHC difference in vACC. Microstructural features of white matter tracts connecting functionally abnormal regions did not reveal any differences between the groups. VMHC values in vACC negatively correlated with the quality of life (QOL) scores of patients. In conclusion, this study provides preliminary evidence of the disrupted functional coordination rather than anatomic coordination between interhemispheric regions within the cortex-thalamus circuit in IBS patients, which could partly account for the enhanced visceral information processing and impaired endogenous pain or emotion inhibition associated with IBS.

## Introduction

Irritable bowel syndrome (IBS), a common functional gastrointestinal disorder with an estimated worldwide prevalence of 10%–20% (Longstreth and Wolde-Tsadik, 1993), is characterized by chronically recurring abdominal pain or discomfort and altered bowel habits (Mayer, 2008; Chey et al., 2015). IBS has a negative impact on quality of life (QOL) and is a healthcare burden on society (Canavan et al., 2014). Elucidating the pathophysiology of IBS could lead to the identification of reliable diagnostic biomarkers and novel therapeutic approaches; however, the exact mechanisms of IBS remain poorly understood.

In the absence of detectable organic causes, IBS is referred to as a functional disorder, thought to result from a dysregulation of the brain-gut interaction (Mayer and Tillisch, 2011; Koloski et al., 2012; Weaver et al., 2016). Functional neuroimaging (resting-state and task response) allows for the quantification of the viscerosensory inputs that reach the brain, together with structural and diffusion tensor imaging (DTI), multimodal brain imaging makes it possible to identify the human brain circuitry that is correlated with various phenotypic and behavioral manifestations of functional gastrointestinal disorders including IBS (Mayer et al., 2009). The most striking neuroimaging findings in IBS to date have been evidence of greater engagement of specific nodes of the emotional arousal network (e.g., amygdala) and homeostatic afferent network (e.g., thalamus and insular), while there was lower activation of regions involved in the modulation of pain, emotion and attention (e.g., medial prefrontal cortex; Posserud et al., 2006; Mayer et al., 2009; Al Omran and Aziz, 2014; Weaver et al., 2016). The majority of the previous studies in IBS investigated brain abnormalities from the aspect of regional activation during visceral distension in task design (Rapps et al., 2008; Mayer et al., 2009). Recently, resting-state functional connectivity (RSFC), a method that measures inter-regional temporal synchronization between a predefined seed region and functionally related regions at resting-state (van de Ven et al., 2004), has been increasingly used as a reliable and sensitive index in various disease populations (Mueller et al., 2012; Castellanos et al., 2013). RSFC alterations have been reported in patients with chronic pain disorders, including fibromyalgia (Jensen et al., 2012), chronic back pain (Baliki et al., 2012) and migraines (Mainero et al., 2011). The few RSFC studies in IBS have also demonstrated disrupted RSFC in the insular (Hong et al., 2014), amygdala (Qi et al., 2016b), salience/executive control network (Gupta et al., 2014) and default mode network (Qi et al., 2016a) in patients, mostly affecting the bilateral hemispheres. The high degree of functional interaction between cerebral hemispheres is a fundamental characteristic of the intrinsic functional architecture of the brain (Zuo et al., 2010). However, little is known about the interhemispheric functional synchronization and its associated anatomic connectivity in IBS.

This study examined abnormalities in the functional coordination between hemispheres in IBS. To do so, we used a novel method called voxel-mirrored homotopic connectivity (VMHC; Zuo et al., 2010) to measure the strength of intrinsic functional connectivity between hemispheres in a voxel-wise manner. We hypothesized that IBS patients would show higher VMHC in the emotional, arousal and homeostatic afferent networks, but lower VMHC in the modulation systems of pain, emotion and attention compared to healthy controls (HCs). We also investigated the corresponding anatomical connections using DTI to reveal whether the changes in interhemispheric functional coordination resulted from alterations in anatomic coordination.

## Materials and Methods

### Patients

The study cohort included 68 IBS patients and 68 HCs; all participants were right-handed and gave written informed consent to the procedures approved by the local Medical Research Ethics Committee of Jinling Hospital in accordance with the Helsinki Declaration. All the participants were volunteers. All patients were recruited from the Digestive Disease Clinic of our hospital and were clinically diagnosed with IBS by a gastroenterologist with expertise in functional gastrointestinal disorders based on the Rome III criteria (Drossman, 2006) and the healthy controls were collected from the local communities. The diagnostic criteria included recurrent abdominal pain or discomfort associated with two or more of the following: relief/improvement by defecation, onset related to a change in stool frequency, and onset related to a change in stool appearance.

Subjects were excluded if they reported a history of gastrointestinal surgery, psychiatric illnesses or substance abuse, treatment with any centrally acting medications such as selective serotonin reuptake inhibitors, aspirin or non-steroidal anti-inflammatory drugs for over 2 weeks before enrollment, major medical or neurological conditions and head motion of more than 1.0 mm in translation or 1.0° in rotation during magnetic resonance imaging (MRI). Two IBS patients and one healthy subject were excluded for excessive head motion, and one IBS patient was excluded for falling asleep in the MR scanner during scanning according to his self-reporting after scanning. The remaining 65 IBS patients (49 men, 16 women, mean age: 34.00 ± 11.82 years) and 67 HCs (51 men, 16 women, mean age: 31.21 ± 10.70 years) were included in the final analysis and were matched for age, sex and education level.

Questionnaires were completed before MR scanning for all participants, including Mini-Mental State Examination (MMSE; Folstein et al., 1983), Montreal Cognitive Assessment (MoCA; Nasreddine et al., 2005) and Zung Self-Rating Anxiety and Depression Scales (SAS; Zung, 1971) and SDS (Zung et al., 1965), respectively). IBS patients were also assessed by the IBS-Symptom Severity Score (IBS-SSS; Francis et al., 1997), IBS-QOL score (Patrick et al., 1998) and the visual analog scale (VAS, 0–100 points with the descriptors “no pain sensation” at 0 and “the most intense pain sensation imaginable” at 100; Price et al., 1994).

### MRI Data Acquisition

Subjects were scanned using a 3 Tesla MR instrument (TIM Trio, Siemens Medical Solutions, Erlangen, Germany). Foam padding was used to minimize head motion for all subjects, who were instructed to rest with their eyes closed, not think of anything in particular, and not fall asleep. First, high-resolution three dimensional *T*_1_-weighted structural images were acquired in sagittal orientation using a magnetization-prepared rapid acquisition gradient-echo sequence (repetition time/echo time [TE] = 2300 ms/2.98 ms, flip angle = 9°, field of view (FOV) = 256 mm^2^ × 256 mm^2^, matrix size = 256 × 256, slice thickness = 1 mm, 191 slices in the sagittal orientation). Second, resting-state fMRI (rs-fMRI) data were obtained using a single-shot, gradient-recalled echo-planar imaging sequence (TR/TE = 2000 ms/30 ms, FOV = 240 mm^2^ × 240 mm^2^, flip angle = 90°, matrix = 64 × 64, voxel size = 3.75 mm^3^ × 3.75 mm^3^ × 4 mm^3^, 30 axial slices aligned along the anterior-posterior commissure, 250 volumes). Then, diffusion tensor images were obtained using a spin echo-based echo planar imaging sequence in contiguous axial planes, including 20 volumes with diffusion gradients applied along 20 non-collinear directions (b = 1000 s/mm^2^) and one volume without diffusion weighting (b = 0 s/mm^2^). Each volume consisted of 30 contiguous axial slices covering the whole brain (TR/TE = 4100 ms/93 ms, FOV = 240 mm^2^ × 240 mm^2^, matrix = 128 × 128, voxel size = 1.8 mm^3^ × 1.8 mm^3^ × 4 mm^3^).

### Data Processing

#### Functional Images

Functional images were preprocessed by using SPM8 software^1^. The first 10 volumes were excluded to ensure steady-state longitudinal magnetization; the remaining 240 images were corrected for temporal differences and head motion. Individual *T*_1_ images were co-registered to the functional images, and then were segmented using the unified segmentation algorithm (Liu et al., 2012) and normalized into the standard Montreal Neurological Institute (MNI) space. Functional images were subsequently warped into the MNI stereotaxic space of 3 mm^3^ × 3 mm^3^ × 3 mm^3^ by applying the parameters of the *T*_1_ image normalization. To account for differences in geometric configuration between hemispheres, functional images were further transformed to a symmetric space under the following procedures: first, normalized *T*_1_ images (in MNI space) of all subjects were averaged with their left–right mirrored version, producing a group-specific symmetrical template; second, the functional images were further normalized to this symmetrical template associated with *T*_1_ images; finally, the functional images were spatially smoothed with an 8 mm full width at half maximum isotropic Gaussian kernel.

After smoothing, images were temporally filtered (bandpass: 0.01 Hz–0.08 Hz), and several sources of spurious variance were regressed out (six head motion parameters, mean signals from cerebrospinal fluid, white matter and the whole brain; Fox et al., 2005). VMHC analysis was performed by using the rs-fMRI Toolkit (REST^2^). Pearson correlations were computed between symmetric voxels in bilateral hemispheres. The resulting correlation between each paired voxel constituted a VMHC brain map (Fisher *z* transformed) and was used for group-level analysis.

#### Diffusion Tensor Images

Diffusion data preprocessing was performed using the Pipeline for Analyzing Brain Diffusion Images toolkit (PANDA^3^; Cui et al., 2013), which synthesizes procedures in FSL^4^ and the Diffusion Toolkit^5^. Individual diffusion images were geometrically corrected using an unweighted B0 image (b = 0 s/mm^2^) and a filed map, and then co-registered to the B0 image with linear least-squares fitting method to minimize head movements. Diffusion-tensor models were estimated at each voxel. Whole-brain fiber tracking was performed in the DTI native space for each subject with a continuous tracking algorithm embedded in the Diffusion Toolkit. Path tracing proceeded until either the fractional anisotropy (FA) fell below 0.15, or the minimum angle between the current and the previous path segment was higher than 35°, as was done in our previous study (Qi et al., 2012).

The regions with abnormal VMHC in IBS patients were selected as regions of interest (ROIs) for DTI data analysis. Fiber bundles connecting symmetrical ROIs in each hemisphere were then extracted from the whole-brain fibers. This was done as follows: first, the ROIs were transformed from the normalized symmetric space to each individual’s native functional space; second, the mean functional image (native functional space) was co-registered to the B0 image (native diffusion space) and this transformation was applied to all ROIs; third, the ROIs were dilated by one voxel into the white matter to ensure they were in contact with the fibers; finally, only those tracts that reached the symmetrical ROIs were picked from the whole-brain fiber tracking. This was accomplished using TrackVis software^6^.

### Statistical Analysis

For each group, a random-effects one-sample *t* test was performed with SPM8^7^ for individual VMHC maps. Significant clusters were identified using the joint expected probability distribution (Poline et al., 1997) with height (*P* < 0.005) and extent (*P* < 0.05) thresholds corrected at the whole-brain level. To assess differences of VMHC between groups, a random two-sample *t* test was then performed, while eliminating the effects of age, sex and educational level by regression. Significance thresholds were set at a corrected *P* < 0.05, with the joint expected probability distribution as done in the abovementioned one-sample *t* test. Path length, tract count and mean FA of the fiber connecting the bilateral ROIs were compared between groups by a two-sample *t* test using SPSS v16.0 (SPSS Inc., Chicago, IL, USA), which was considered significant at *P* < 0.05. The comparison between the IBS and control groups was also performed by including anxiety and depression as covariates (Zhou et al., 2013) to evaluate the effect of anxiety and depression on interhemispheric coordination during two-sample *t* test. Since the SAS and SDS scores manifested a high correlation here (patient group: Spearman rho = 0.77, *P* < 0.001; control group: Spearman rho = 0.69, *P* < 0.001), they were taken together, rather than separately, as covariates to evaluate the psychosocial effect on brain connectivity as done in previous studies in IBS (Zhou et al., 2013; Qi et al., 2015).

To investigate the relationship between abnormal interhemispheric connectivity and clinical indications of IBS, any functional and structural connectivity that differed significantly between IBS patients and HCs was extracted and correlated with IBS-SSS, IBS-QOL, SAS, SDS and pain intensity scores of IBS patients using Pearson’s correlation analysis, which was considered significant at *P* < 0.05.

## Results

### Clinical Data

The demographic and clinical data are summarized in Table 1. There were no differences in age, sex or education level between groups (*P* > 0.05, all). Scores for MMSE (>26) and MoCA (≥26) for all subjects were in the normal range, while lower MMSE and higher anxiety and depression symptom scores were observed in IBS patients relative to HCs (*P* < 0.05; Table 1). All cases were diarrhea-predominant based on bowel habits, with pain reported as a symptom by each IBS patient.

**Table 1:** **Demographic and clinical data for IBS patients and HCs**.

Protocols	HC (*n* = 67)	Patients (*n* = 65)	*P* value	χ^2^/*t* value
Sex (M/F)	51/16	49/16	0.92^a^	0.01
Age (±SD), years	31.21 ± 10.70	34.00 ± 11.82	0.16^b^	1.4
Education, years	14.46 ± 3.22	13.29 ± 4.70	0.10^b^	−1.7
SAS	33.59 ± 4.49	41.85 ± 9.64	<0.001^b^	4.0
SDS	37.11 ± 7.61	42.29 ± 9.82	0.001^b^	3.4
MMSE	29.39 ± 1.03	36.81 ± 2.85	<0.001^b^	19.9
MoCA	27.66 ± 2.35	26.81 ± 3.34	0.17^b^	−1.3
IBS-QOL		69.77 ± 23.26		
IBS-SSS		179.00 ± 68.48		
VAS (pain) intensity		32.77 ± 21.45		
During (months)		42.11 ± 48.34		

*Values are expressed as mean ± standard deviation (range). ^a^P value for sex distribution was obtained by the *χ*^2^ test. ^b^P values for age, education and neuropsychological test scores were obtained by the two-sample t test. IBS, irritable bowel syndrome; HC, healthy control; SAS, Self-Rating Anxiety Scale; SD, Self-Rating Depression Scale; MMSE, Mini-Mental State Examination; MoCA, Montreal Cognitive Assessment; IBS-QOL, IBS-Quality of Life; IBS-SSS, IBS-Symptom Severity Score; VAS, visual analog scale*.

### Interhemispheric Functional Connectivity

**Figure 1:**
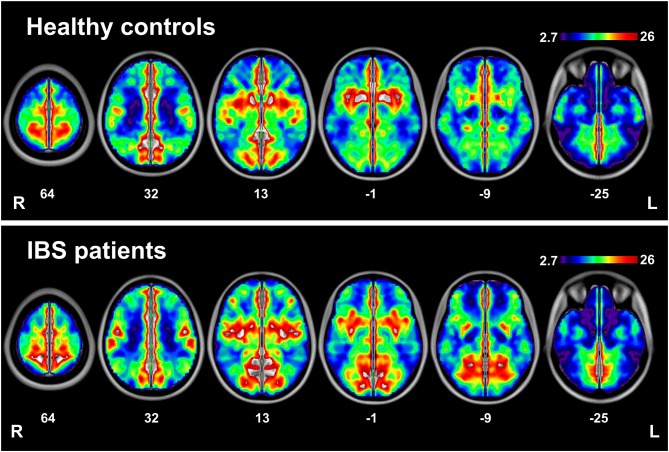
**Interhemispheric functional connectivity within each group.** Regions show significant interhemispheric functional connectivity in healthy controls (HCs) and patients with irritable bowel syndrome (IBS), respectively.

**Figure 2:**
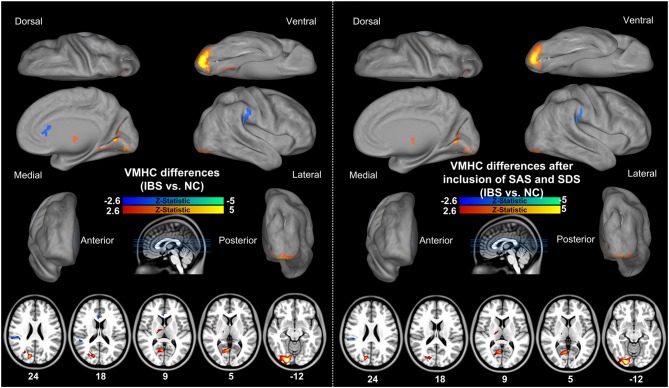
**Group comparison of interhemispheric functional connectivity between IBS patients and HCs.** Results of a two-sample *t*-test of VMHC reveal lower VMHC in vACC and IPL, while higher VMHC in thalamus, cuneus, PCC, lingual gyrus and inferior occipital/cerebellum lobes in IBS patients relative to HCs. Inclusion of anxiety and depression as covariates eliminated the VMHC difference in the vACC between two groups. IBS, irritable bowel syndrome; VMHC, voxel-mirrored homotopic connectivity; vACC, ventral anterior cingulate cortex; IPL, inferior parietal lobule; PCC, posterior cingulate cortex.

Figure 1 shows the spatial patterns of VMHC in each group. Visual inspection indicated that the functional coordination was a global brain phenomenon, with regional differences in strength consistent with previous studies (Zuo et al., 2010; Ji et al., 2014; Li et al., 2015). The HC and IBS groups did not differ in global VMHC (IBS patients: 0.64 ± 0.08; controls: 0.63 ± 0.07; *t* = 0.67, *P* = 0.50); however, inter-group comparisons showed that IBS patients had lower VMHC in bilateral ventral anterior cingulate cortices (vACC) and inferior parietal lobules (IPL), as well as higher VMHC in bilateral thalami, cuneus, posterior cingulate cortices (PCC), lingual gyri and inferior occipital/cerebellum lobes relative to HCs (Table 2; Figure 2). Inclusion of anxiety and depression as covariates (along with age, sex and education level) eliminated the VMHC difference in the vACC (Figure 2).

**Table 2:** **Brain regions showing VMHC differences between IBS patients and HCs**.

Brain regions	<*P* value	Cluster Size (voxels)	BA	Maximal *T* score^#^	Primary peak location (*x, y, z*)
ventral ACC	0.032	40	24	−2.76	±6, 24, 18
IPL	0.002	96	40	−2.63	±60, -36, 33
Thalamus	0.036	38		±3.17	±18, -15, 9
Inferior occipital/Cerebellum lobes	<0.001	241	18,19	+4.36	±24, -87, -12
Cuneus	<0.001	67	18	+4.81	±15, -81, 21
PCC	<0.001	48	30	+4.41	±6, -63, 6
Lingual gyrus	<0.001	158	30	+3.43	±12, -63, 6

*^#^Positive and negative values represent higher and lower VMHC, respectively. VMHC, voxel-mirrored homotopic connectivity; IBS, irritable bowel syndrome; BA, Brodmann’s area; MNI, Montreal Neurological Institute; ACC, anterior cingulate cortex; IPL, inferior parietal lobule; PCC, posterior cingulate cortex*.

### Interhemispheric Anatomical Connectivity

**Figure 3:**
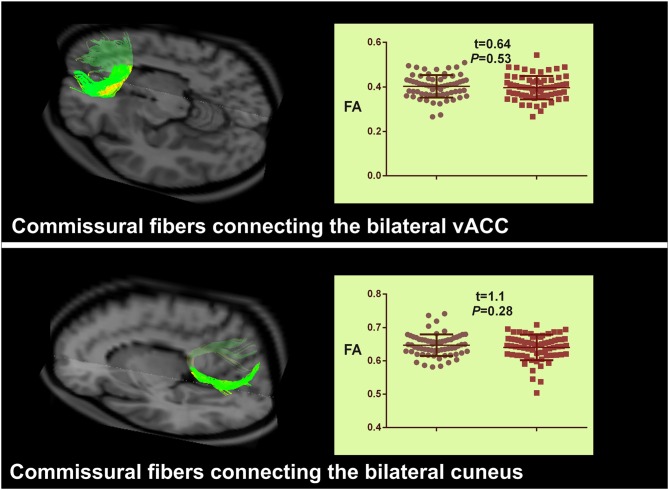
**Group comparison of interhemispheric anatomical connectivity between IBS patients and HCs.** Commissural fibers connecting the bilateral vACC, and cuneus are illustrated by the diffusion tractographic image from a single control subject. The structural features of these two tracts do not show any significant differences between groups. IBS, irritable bowel syndrome; vACC, ventral anterior cingulate cortex.

Two commissural fibers connecting the left and right vACC and cuneus were detected in all HCs and patients. The commissural fibers that connected the other bilateral ROIs displaying abnormal VMHC were detected in less than half of the subjects. Therefore, we only extracted the anatomical parameters in commissural fibers connecting the bilateral vACC and cuneus. The result of a two-sample *t* test between groups did not show any significant differences in path length, tract count or FA of the two fibers (Figure 3).

### Correlation Analysis

**Figure 4:**
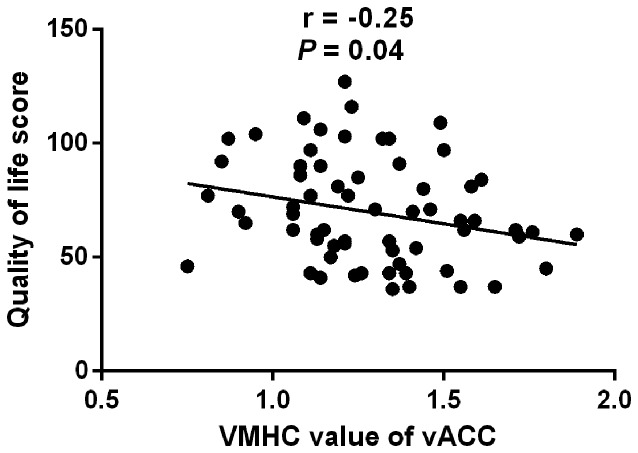
**Correlation between VMHC in vACC and quality of life score in IBS patients.** The VMHC values of bilateral vACC show a slight negative correlation with the QOL scores of IBS patients (*r* = −0.25, *P* = 0.04) IBS, irritable bowel syndrome; VMHC, voxel-mirrored homotopic connectivity; vACC, ventral anterior cingulate cortex; QOL, quality of life; A.U., arbitrary unit.

The VMHC value in vACC showed a slight negative correlation with the QOL scores of IBS patients (*r* = −0.25, *P* = 0.04; Figure 4). The VMHC value in other regions had no significant correlation with clinical indices in IBS patients.

## Discussion

In this study, we investigated interhemispheric functional and anatomic coordination in IBS patients by combining rs-fMRI and DTI. We demonstrated specific disruptions of functional coordination rather than anatomic coordination in IBS patients compared with HCs. The VMHC abnormalities in IBS mainly localized in the cortex-thalamus circuit, with higher VMHC values in thalamus, cuneus, PCC, lingual gyrus and inferior occipital/cerebellum lobes, and lower values in vACC and IPL. Moreover, the inclusion of anxiety and depression as confounding variables led to a loss of inter-group VMHC differences in the vACC. On the contrary, anatomical connectivity showed no significant difference between groups.

### Higher Interhemispheric Functional Connectivity in IBS

Our results showed that IBS patients had higher interhemispheric functional connectivity of bilateral thalami. The thalamus is a core region of the homeostatic afferent network, which encompasses the sensory input entering the thalamus from the brainstem, and then projects to the insular and anterior mid-cingulate cortex, mediating affective, motivational and motor aspects of the stimulus (Al Omran and Aziz, 2014). Convergent evidence has suggested that the thalamus was more activated in IBS patients than the HCs during visceral distension (Ringel et al., 2003; Yuan et al., 2003) and cutaneous heat stimuli (Verne et al., 2003). In a recent rs-fMRI study using regional homogeneity (ReHo), an indicator measuring the degree of regional synchronization of fMRI time series (Zang et al., 2004), Ke et al. (2015) reported higher regional synchronization in the thalamus of IBS patients relative to controls. The ReHo in that study and VMHC used in this study focus on the degree of regional- and interhemispheric-coordination, respectively. Thus, the finding of a higher VMHC of the thalamus in IBS was in accordance with previous findings and added important insights into understanding the role of thalamus in enhanced visceral information processing of IBS.

The cuneus and PCC also showed higher VMHC in IBS patients. The cuneus plays an important role in integrating somatosensory inputs with other sensory stimuli and with cognitive processes such as attention, learning and memory (Fulbright et al., 2001). It is also considered to be the center of the cortical network associated with the experience of pain (Fulbright et al., 2001). One recent rs-fMRI study also demonstrated increased intrinsic activity of the bilateral cuneus in IBS, which was regarded as partly accounting for the chronic pain of the disease (Qi et al., 2015). PCC is involved in internally directed cognition, conscious awareness and working memory (Leech and Sharp, 2014). Previous studies have reported higher PCC activation in IBS patients (Naliboff et al., 2001; Verne et al., 2003), and even higher in those with a history of sexual or physical abuse (Ringel et al., 2008). However, it is worth noting that a subsequent study showed decreased intrinsic brain activity in the PCC (Qi et al., 2015). These discrepancies may be explained by the use of different methodologies. The previous study that showed decreased PCC activity applied the amplitude of low-frequency fluctuation (ALFF) algorithm, an index evaluating the strength or intensity of spontaneous neural activity at rest (Zang et al., 2007), while the VMHC used here measures the strength of interregional temporal correlation (functional connectivity) between hemispheres.

We also found higher VMHC in the lingual gyrus and inferior occipital/cerebellum in IBS patients. Few studies have reported abnormalities in these regions in IBS patients. Ke et al. (2015) showed increased regional synchronization of cerebellum regions in IBS; however, the consistency of these findings needs to be confirmed in future studies.

### Lower Interhemispheric Functional Connectivity in IBS

Our finding that decreased interhemispheric functional connectivity in the vACC was shown for IBS patients, but the inclusion of anxiety and depression as covariates abolished this difference. The ACC is a multifunctional structure situated in the medial frontal lobe that is highly interconnected with the insular, prefrontal, limbic and other subcortical structures, thus cognitive and affective factors may exert influence on pain transmission through the ACC (Bush et al., 2000; Vogt, 2005). The vACC is the principal site of autonomic (primarily vagal) regulation in the frontal lobe and plays a key role in the visceral aspects of emotion (Vogt, 2005; Shackman et al., 2011). Recent studies have revealed that the symptoms of anxiety and depression partially mediated the central processing of visceral stimuli in patients with IBS (Elsenbruch et al., 2010), and abolished differences in multiple white matter tracts between patients with functional gastrointestinal disorders and HCs (Zhou et al., 2013). A recent fMRI study also reported that the inclusion of anxiety and depression as covariates abolished the decreased ALFF of ACC in IBS patients (Qi et al., 2015). Thus, our findings are supported by previous neuroimaging studies and indicate that the high level of depression and anxiety may lead to affective dysregulation of the ACC in IBS patients. Together with previous studies (Wilder-Smith et al., 2004; Ringel et al., 2008), we conclude that ACC might be the key area associated with affective dysregulation in IBS.

In addition to decreased VMHC in the vACC, lower VMHC in the IPL was also observed in IBS patients relative to controls. The IPL is also implicated in pain modulation (Schwedt and Chong, 2014). A previous study demonstrated lower spontaneous brain activity in bilateral inferior parietal cortices in IBS patients (Ke et al., 2015); therefore, our results are compatible with previous neuroimaging studies in IBS.

### Interhemispheric Anatomical Connectivity in IBS

We performed an inter-group analysis for the commissural tracts connecting the bilateral ACC and cuneus, but did not observe significant differences between groups. Some DTI studies in IBS have demonstrated abnormalities of microstructural white matter integrity (Chen et al., 2011; Ellingson et al., 2013). For instance, Chen et al. (2011) reported that IBS patients had an increased FA in the fornix and the external capsule compared with HCs. Ellingson et al. (2013) also found IBS patients had lower FA in thalamic regions, the basal ganglia and sensory/motor association/integration regions as well as higher FA in frontal lobe regions and the corpus callosum. However, across these investigations that employ DTI, no consistent areas of abnormal microstructural white matter were reported. The value of directly comparing our findings with previous DTI studies was limited by the differences in study samples, analysis methodology (measurements of the microstructure of regional white matter in most previous studies and of the whole fiber tract that connects cerebral hemispheres in the current study), as well as the mixed findings within the previous DTI studies.

Taking into account that IBS patients showed abnormal interhemispheric functional connectivity but no significant changes in interhemispheric anatomic connectivity, we speculated that as for functional disorders such as chronic pain disorders including IBS, functional connectivity may be more sensitive than structural connectivity in detecting the exact mechanisms underlying the symptoms. In general, the functional connectivity which measures blood oxygenation level-dependent fluctuations is thought to be more flexible and sensitive; while the structural connectivity is relatively stable (Bullmore and Sporns, 2009; Zhang et al., 2011). The inconsistent changes of interhemispheric functional and structural coordination have also been reported in other brain disorders without significant lesions by conventional MRI. For instance, Wu et al. (2015) reported widespread impaired interhemispheric functional connectivity but no affected interhemispheric structural connectivity in benign childhood epilepsy with centrotemporal spikes.

While this study has advanced our understanding of the changes in neural networking associated with IBS, it also had some limitations. First, our study cohort consisted of Chinese patients with diarrhea-predominant IBS, and as such, the results may not be generalizable to patients of other ethnicities or with other subtypes of IBS. Second, future studies will need to address the question of whether the observed results are altered by IBS treatment. Besides, further studies with more subjects with depression or anxious symptom and with different pain degrees are needed to investigate how the psychosocial factor and pain might modulate the observed differences. Third, as a newly developed approach, the exact physiological implication of VMHC and its relationship with anatomical connectivity warrant further examination.

## Conclusion

Our data provide preliminary evidence of the potential value of VMHC and DTI in detecting abnormalities of interhemispheric coordination in IBS patients. We demonstrated disrupted interhemispheric functional coordination rather than anatomical coordination in IBS patients, mainly localized within the cortex-thalamus circuit, which could partly explain the enhanced visceral information processing and impaired endogenous pain or emotion regulations associated with IBS.

## Author Contributions

RQ was involved in literature review, experimental design, data analysis and writing of the manuscript. CL contributed to the data collection and the analysis of neuropsychological data. YW and LC contributed to the data collection. QX was involved in fMRI data analysis. FW contributed in the experimental design. LJZ contributed in the experimental design and revision of the manuscript. GML was involved in the experimental design and revision of the manuscript.

## Conflict of Interest Statement

The authors declare that the research was conducted in the absence of any commercial or financial relationships that could be construed as a potential conflict of interest.

## Footnotes

^1^http://www.fil.ion.ucl.ac.uk/spm

^2^http://www.restfmri.net

^3^http://www.nitrc.org/projects/panda

^4^http://fsl.fmrib.ox.ac.uk/fsl/

^5^http://www.trackvis.org/dtk/

^6^www.trackvis.org

^7^http://www.fil.ion.ucl.ac.uk/spm
